# A Dual-Mode “Turn-On” Ratiometric Luminescent Sensor Based on Upconverting Nanoparticles for Detection and Differentiation of Gram-Positive and Gram-Negative Bacteria

**DOI:** 10.1021/acsomega.5c07006

**Published:** 2025-09-23

**Authors:** Marylyn S. Arai, Gabriel V. Brambilla, Bruna Carolina Corrêa, Leonnam G. Merízio, Natalia M. Inada, Andrea S. S. de Camargo

**Affiliations:** † 117186 São Carlos Institute of Physics, University of São Paulo,13566-590 São Carlos, Brazil; ‡ 42220 Federal Institute for Materials Research and Testing (BAM), 12489 Berlin, Germany; § Otto Schott Institute of Materials Research, Friedrich Schiller University, 07743 Jena, Germany; ∥ Department of Biomedical Engineering, Texas A&M University, College Station, Texas 77843, United States

## Abstract

Infectious bacterial diseases, intensified by antibiotic resistance, cause millions of deaths annually and pose risks beyond human health, including water and food contamination. Current diagnostics are often slow, require complex equipment, and lack specificity, highlighting the need for rapid and reliable detection methods. To address this, we developed a luminescent sensor based on NaYF_4_ upconverting nanoparticles (UCNPs) doped with Er^3+^ or Tm^3+^, coated with COOH-PEG_4_-COOH, and functionalized with vancomycin (Van) or polymyxin-B (Poly) to selectively target Gram-positive and Gram-negative bacteria, respectively. Gold nanoparticles (AuNPs) served as quenchers, enabling a ratiometric “turn-on” mechanism: upon bacterial binding, the UCNP emission, initially quenched by AuNPs, was partially restored. This allowed differentiation through changes in the green/red (G/R) ratio for Er-UCNP@PEG_4_-Van and the blue/red (B/R) ratio for Tm-UCNP@PEG_4_-Poly. The sensor distinguished between Gram-positive and Gram-negative bacteria over a wide concentration range (0.05 to 5 × 10^5^ CFU/mL) and showed high correlation with actual bacterial counts (*r* = 0.99 for *S. aureus*, *r* = 0.91 for *E. coli*). This platform is a potential fast, selective, and reliable tool for bacterial detection in clinical and environmental settings.

## Graphical abstract

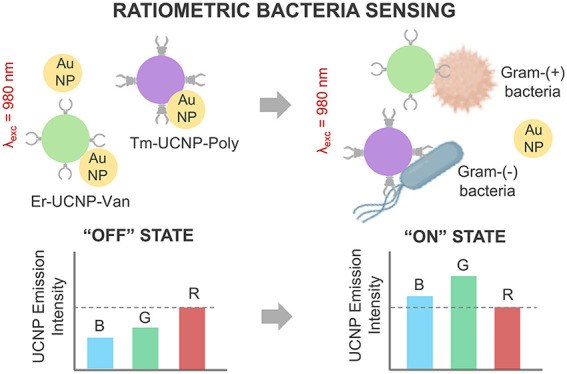

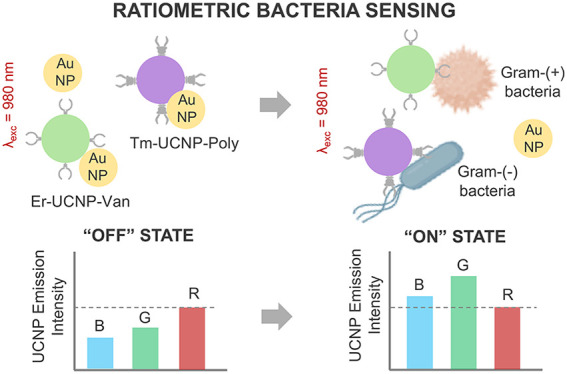

## Introduction

Infectious bacterial diseases remain a significant global health challenge, causing approximately 17 million deaths annually and accounting for one-third of global mortality. The rapid emergence of antibiotic-resistant bacteria has exacerbated this crisis, making infections increasingly difficult to diagnose and treat and thus leading to thousands of deaths each year, numbers that rival the combined fatalities from AIDS, tuberculosis, and viral hepatitis.^1,2^ Projections indicate that by 2050, more than 10 million people could die annually from infections caused by resistant microorganisms, surpassing mortality rates from diseases such as cancer and diabetes.^3^

Beyond human infections, bacterial contamination of water and food supplies presents a serious public health risk, leading to widespread outbreaks of diseases, economic losses, and long-term environmental damage. Contaminated water and food can harbor a range of pathogenic bacteria that are responsible for illnesses such as gastroenteritis, cholera, and typhoid fever.^4–6^ Recent advancements in bacterial detection have led to the development of a range of identification strategies aimed at differentiating Gram-positive and Gram-negative bacteria. While Gram staining is a well-established and widely used method for bacterial classification, it provides only qualitative information and requires microscopy infrastructure. New methods have been constantly developed, including colorimetric and lateral flow assays, which offer visual readouts but often lack sensitivity and quantitative capability; PCR-based techniques, which provide high specificity but require complex equipment and trained personnel; electrochemical biosensors, which offer promising sensitivity but are prone to interference from complex sample matrices; and fluorescence/luminescence-based sensors, which provide real-time detection but often rely on organic dyes susceptible to photobleaching. Despite these efforts, many existing methods still struggle to balance speed, specificity, sensitivity, and ease of use, particularly in point-of-care or field settings. Therefore, there remains a clear need for robust, selective, and rapid detection platforms capable of accurately distinguishing between Gram-positive and Gram-negative bacteria under simple experimental conditions.^7,8^

In this context, upconverting nanoparticles (UCNPs) have shown great promise in the development of luminescent-based sensors for bacterial detection.^9^ The UCNPs offer several unique advantages that make them highly attractive for biosensing applications. Their excitation in the near-infrared (NIR) region, typically at 980 nm, significantly reduces background autofluorescence from biological samples, minimizes photodamage to cells and tissues, and allows for deeper optical penetration, features that are particularly advantageous for bioimaging and biosensing in complex media. Unlike traditional organic fluorophores, UCNPs possess a highly stable inorganic crystal lattice that resists photobleaching and chemical degradation.^10,11^ Moreover, they exhibit sharp, well-defined emission bands and can be engineered to emit multiple colors simultaneously, enabling ratiometric measurements with enhanced accuracy and built-in self-calibration. These characteristics make UCNPs a powerful platform for the development of robust, sensitive, and selective luminescent sensors.^12,13^

Previous research has demonstrated the use of UCNP-based sensors for detecting specific bacteria, such as methicillin-resistant *Staphylococcus aureus* (MRSA) DNA, and for identifying *Escherichia coli* and *Salmonella typhimurium*, achieving impressive LODs.^14–16^ However, most existing UCNP-based sensors are highly specific to a single type of bacteria and often require sophisticated instrumentation, limiting their practical applications in broader contexts. To address and overcome these limitations, our work focuses on developing a novel UCNP-based luminescent sensor capable of selectively detecting Gram-positive and Gram-negative bacteria. This distinction is clinically relevant, as it enables a more accurate selection of antibiotic treatments based on the bacterial classification.

Our proposed sensor takes advantage of the selective binding capabilities of the antibiotics vancomycin (Van) and polymyxin-B (Poly), which target Gram-positive and Gram-negative bacteria, respectively. Van, known for its affinity to the d-alanyl-d-alanine termini of the cell wall peptidoglycan in Gram-positive bacteria,^17,18^ and Poly, which binds to lipopolysaccharides in the outer membrane of Gram-negative bacteria,^19,20^ were conjugated to the surface of UCNPs. This conjugation was achieved by coating the UCNPs with poly­(ethylene glycol) diacid (COOH-PEG_4_-COOH), followed by the attachment of Van to Er-doped UCNPs (Er-UCNP@PEG_4_-Van) and Poly to Tm-doped UCNPs (Tm-UCNP@PEG_4_-Poly). The core mechanism of the sensor is based on the ratiometric “turn-on” approach, which involves the initial UCNP emission quenching by gold nanoparticles (AuNPs) and subsequent partial recovery of UCNP emissions upon bacterial binding. It operated by monitoring the changes in the green/red (G/R) and blue/red (B/R) ratios of Er^3+^ and Tm^3+^ emissions, respectively. The G/R ratio is primarily used to detect Gram-positive bacteria, with the green emission from Er-UCNP@PEG_4_-Van increasing upon target binding, while the B/R ratio is used to detect Gram-negative bacteria, with the blue emission from Tm-UCNP@PEG_4_-Poly increasing upon interaction with the target microorganism.

This work involved a comprehensive study of the key stages required to produce the proposed sensor, including (1) the optimized synthesis of highly luminescent UCNPs; (2) the coating of UCNPs and posterior functionalization with antibiotics; (3) the synthesis of gold nanoparticles with an optimized surface plasmon resonance (SPR) absorption band; and (4) the development and optimization of the ratiometric “turn-on” sensor. The results demonstrate the effectiveness of this platform in detecting and distinguishing between Gram-positive and Gram-negative bacteria within a wide concentration range, from 0.05 to 5 × 10^5^ CFU/mL. The sensor was further validated by testing spiked samples, where it showed an excellent correlation between the measured and actual bacterial concentrations for both *Staphylococcus aureus* (*r* = 0.99) and *Escherichia coli* (*r* = 0.91), which were used as model Gram-positive and Gram-negative bacteria, respectively. By combining the selective binding of antibiotics with the unique optical properties of UCNPs, our sensor provides a reliable, innovative method for detecting and differentiating bacteria, offering a valuable tool for rapid diagnostics in environmental and clinical settings with potential for miniaturization and automation, which could be advantageous in point-of-care or resource-limited settings.

## Results and Discussion

### Sensing Principle

The produced sensor can detect, differentiate, and quantify the presence of Gram-positive and Gram-negative bacteria in aqueous media. The core concept of the platform, schematically shown in Figure 1, revolves around the ratiometric “turn-on” mechanism, where the UCNPs’ green and blue emissions, initially quenched by the presence of AuNPs, are partially restored upon specific interaction with the targeted bacteria. To achieve this, Er-UCNPs and Tm-UCNPs were selectively functionalized with antibiotics: vancomycin for Gram-positive bacteria and polymyxin-B for Gram-negative bacteria, respectively.

**Figure 1:**
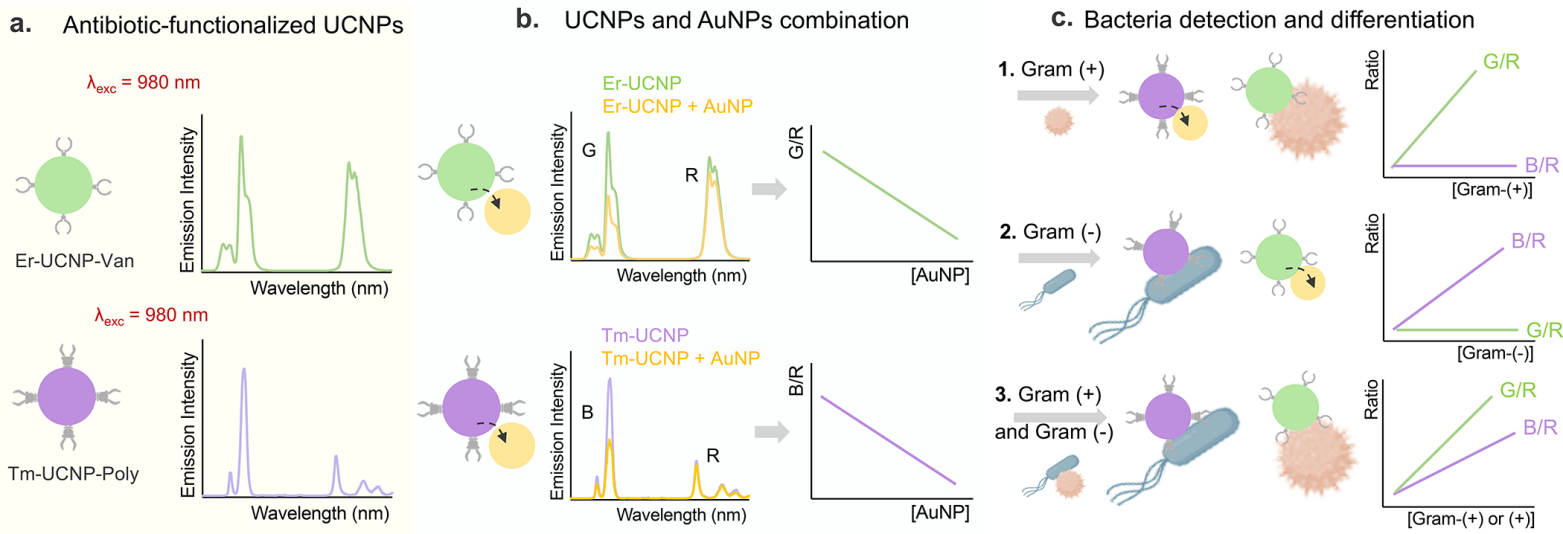
Mechanism of bacteria detection using the developed UCNP-based ratiometric sensor. (a) Schematic representation of antibiotic-functionalized UCNPs: Er-UCNP-Van and Tm-UCNP-Poly and their emission spectra under 980 nm excitation. (b) Combination of UCNPs with AuNPs, the quenching effect on emission intensity, and the corresponding G/R and B/R ratios as a function of AuNP concentration. (c) Bacteria detection and differentiation mechanism: 1. Detection of Gram-positive bacteria, where the G/R ratio increases and B/R remains constant. 2. Detection of Gram-negative bacteria, where the B/R ratio increases and G/R remains constant. 3. Detection of Gram-positive and Gram-negative bacteria shows changes in both G/R and B/R ratios.

The UCNPs functionalized with antibiotics are brought into proximity with the AuNPs, where the strong surface plasmon resonance of the metallic particles is responsible for the partial quenching of UCNPs’ luminescence emission (Figure 1b). Since the SPR band has a maximum between 500 and 600 nm, the UCNPs’ green (G) and blue (B) emissions are more quenched than the red (R) emission. Consequently, the ratios between the green/red (G/R, for Er-UCNPs) and blue/red (B/R, for Tm-UCNPs) emissions are used to monitor the system’s response, establishing this quenched state as a baseline “off” signal for the sensor.

When these particles are introduced to a sample containing bacteria, the antibiotics on the UCNPs bind specifically to their target microorganism. This binding event causes a physical separation of the UCNPs from the AuNPs due to the formation of a bacterial-antibiotic complex. As the concentration of bacteria in the medium increases, the quenching effect diminishes, and depending on the type of microorganism, the UCNPs’ green or blue luminescence is partially restored, resulting in a ratiometric “turn-on” signal (Figure 1c). An increase in the G/R correlates with the presence of Gram-positive bacteria, while a B/R enhancement indicates the existence of Gram-negative bacteria in the sample. The sensor’s design provides an innovative, fast, and easy-to-use method for detecting, differentiating, and quantifying bacteria, utilizing the unique optical properties of UCNPs and the selective binding capabilities of antibiotics. The ratiometric approach enhances the reliability and accuracy of the sensor’s response, making it a promising tool for rapid bacterial detection in various media.

### UCNP Probe Design and Characterization

The NaYF_4_:18%Yb^3+^,2%Er^3+^@NaYF_4_ and NaYF_4_:25%Yb^3+^,0.3%Tm^3+^@NaYF_4_ nanoparticles were synthesized via the high-temperature coprecipitation method. The synthesis involved the formation of a core–shell structure, where the undoped NaYF_4_ shell enhances upconversion (UC) emission by reducing surface defects and shielding the active lanthanides from nonradiative decay pathways.

A schematic representation of the UCNP energy levels relevant to this work is presented in Figure 2a. In these particles, Yb^3+^ acts as a sensitizer, absorbing most of the energy and transferring it to Er^3+^ or Tm^3+^, the emission centers. Upon 980 nm excitation, the Er-UCNPs showed characteristic bands in the green and red spectral regions, while Tm-UCNPs exhibited emissions in the blue and red regions. Specifically, Er^3+^ ions show strong emissions at approximately 520 and 540 nm, corresponding to the ^2^H_11/2_,^4^S_3/2_ → ^4^I_15/2_ transitions, and in the red region (650 nm), corresponding to the ^4^F_9/2_ → ^4^I_15/2_ transition (Figure 2b). Tm^3+^ ions exhibit emissions at approximately 350 and 360 nm, assigned to the ^1^I_6_ → ^3^F_4_, and ^1^D_2_ → ^3^H_6_ transitions; in the blue region at 450 and 475 nm, corresponding to the ^1^D_2_ → ^3^F_4_ and ^1^G_4_ → ^3^H_6_, transitions; and in the red region at 650 and 690 nm, related to the ^1^G_4_ → ^3^F_4_ and ^3^F_3_ → ^3^H_6_ transitions (Figure 2c).^21^

**Figure 2:**
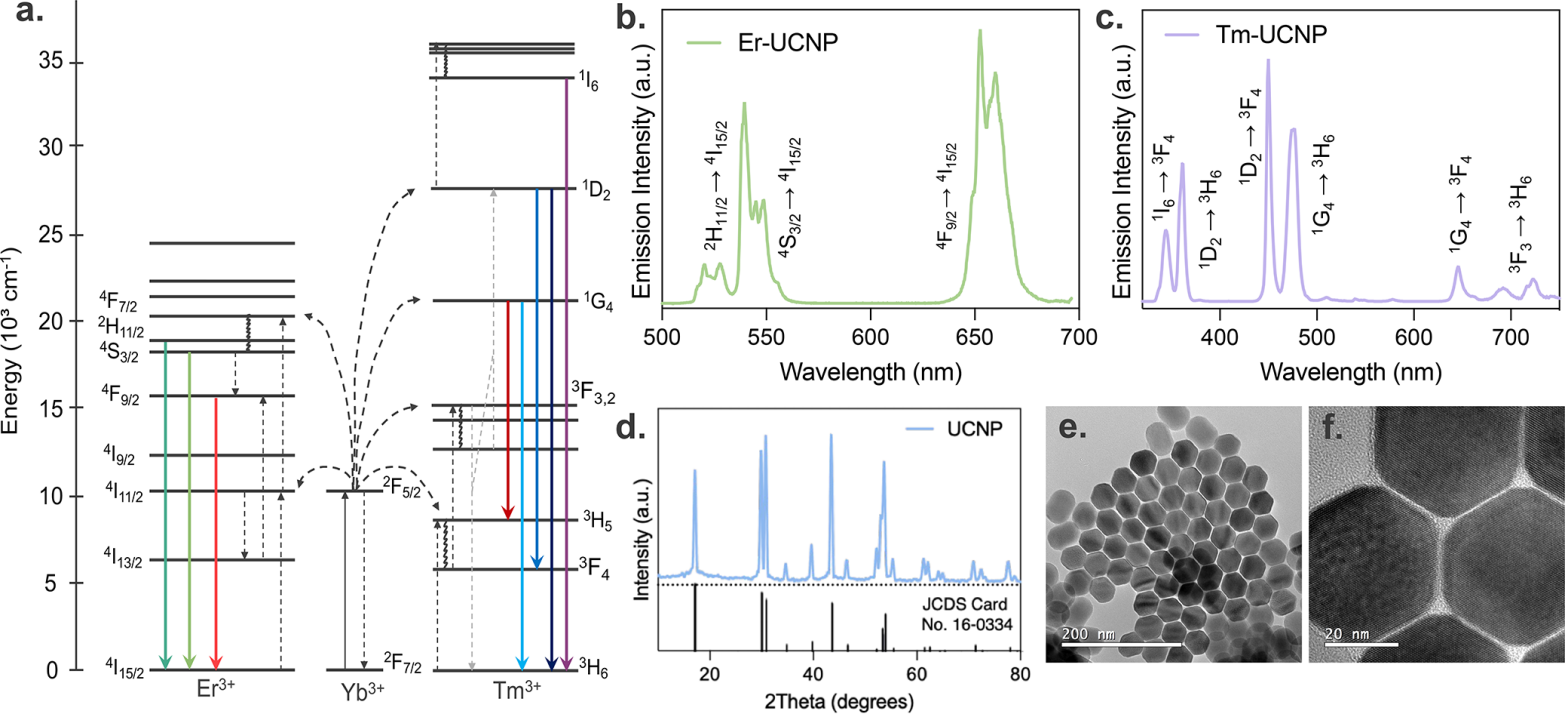
UCNP characterization. (a) Schematic energy level diagram for Er^3+^, Yb^3+^, and Tm^3+^ ions showing the upconversion process. The arrows indicate the energy transfer and emission processes. Emission spectra (λ_exc_ = 980 nm, 500 mW) of (b) Er-UCNPs and (c) Tm-UCNPs in powder. (d) XRD pattern of the prepared UCNPs and reference pattern of the hexagonal (β) NaYF_4_ (JCPDS, no. 16-0334). (e) TEM and (f) high-resolution TEM (HRTEM) images of UCNPs.

The XRD analysis confirmed that the synthesized UCNPs (both Er^3+^- and Tm^3+^-doped) possess a pure hexagonal phase (JCPDS, no. 16-0334) of the NaYF_4_ crystal structure. The diffraction peaks at 2θ values correspond well with the standard hexagonal phase NaYF_4_, indicating high crystallinity and phase purity (Figure 2d). Transmission electron microscopy (TEM) images revealed that the synthesized UCNPs are approximately 40 nm in size and have a uniformly distributed prism hexagonal shape, as shown in Figure 2e,f.

### Antibiotic Conjugation to the Nanoparticle Surface

To combine the antibiotics vancomycin and polymyxin-B with Er-UCNPs and Tm-UCNPs, respectively, the as-prepared hydrophobic UCNPs, coated with oleic acid (UCNP@OA), underwent a ligand exchange reaction where the OA was substituted by the molecule COOH-PEG_4_-COOH (see Materials and Methods) to produce the aqueously stable UCNP@PEG_4_-COOH. The UCNP@PEG_4_-COOH benefits from the hydrophilic nature of PEG, which enhances biocompatibility and reduces nonspecific binding, improving the sensor’s biocompatibility and reducing background noise in biological samples. The flexible PEG chains also allow for a dense and uniform distribution of functional groups, facilitating efficient conjugation with antibiotics. The carboxyl groups (−COOH) at the end of the poly­(ethylene glycol) diacid were then activated and coupled to the amines present in the Van and Poly molecules using the EDC/NHS coupling reaction to produce Er-UCNP@PEG_4_-Van and Tm-UCNP@PEG_4_-Poly, as schematically shown in Figure 3a.

**Figure 3:**
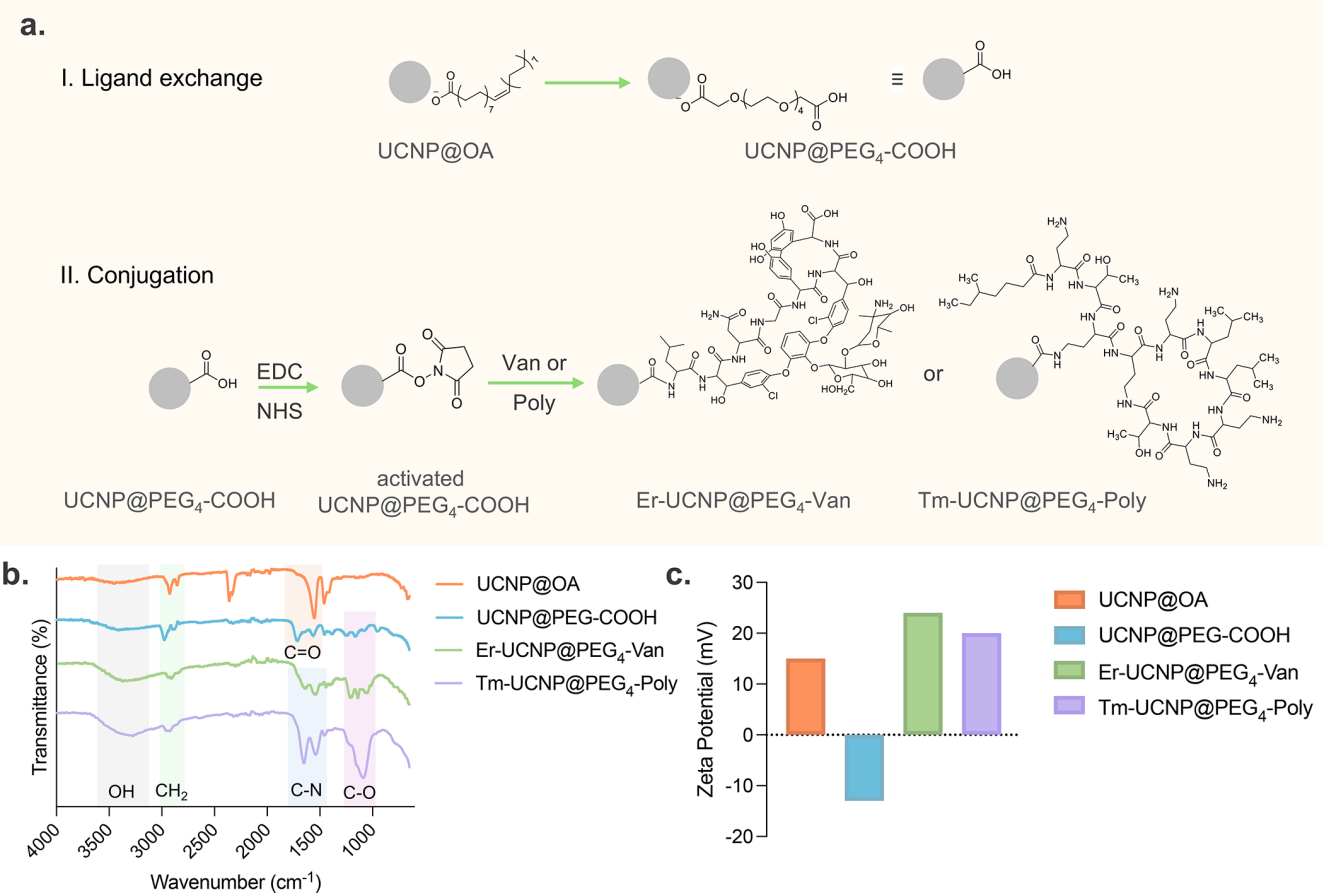
UCNP functionalization. (a) Schematic illustration of the functionalization process. I. Ligand exchange: UCNP@OA is converted to UCNP@PEG_4_-COOH through a ligand exchange reaction. II. Conjugation: UCNP@PEG_4_-COOH is further conjugated with antibiotics (vancomycin or polymyxin-B) using EDC/NHS chemistry, resulting in Er-UCNP@PEG_4_-Van and Tm-UCNP@PEG_4_-Poly. (b) FTIR spectra of different stages of functionalization showing characteristic peaks corresponding to the surface modifications. (c) Zeta potential measurements confirm the surface charge changes after each functionalization step.

Fourier-transform infrared spectroscopy (FTIR) provided clear evidence of the functionalization process and confirmed the successful ligand exchange and antibiotic addition, as shown in Figure 3b. For UCNP@OA, the characteristic peaks of oleic acid, such as the C–H stretching vibrations at around 2924 and 2854 cm^–1^ and the C=O stretching vibration at around 1712 cm^–1^, are observed. After the ligand exchange to form UCNP@PEG_4_-COOH, new peaks appear at around 1100 cm^–1^, which can be attributed to the C–O–C stretching vibrations of the PEG moiety, and a peak appears at around 1720 cm^–1^, which corresponds to the carboxyl group, confirming the successful exchange. Additional changes in the FTIR spectra are observed upon conjugation with vancomycin and polymyxin-B. For Er-UCNP@PEG_4_-Van, new peaks at around 1540 and 1650 cm^–1^ appear, which are characteristic of the amide II and amide I bands, respectively, indicating the presence of amide bonds formed between the carboxyl groups on PEG and the amine groups on Van. Similarly, for Tm-UCNP@PEG_4_-Poly, peaks at around 1540 and 1650 cm^–1^ are also observed, confirming the successful conjugation of polymyxin-B to the UCNPs.

The zeta potential values changed from +15 mV for oleic acid-capped UCNPs to −13 mV for UCNP@PEG_4_-COOH, indicating the successful exchange of oleic acid with PEG. Upon conjugation with Van and Poly, the zeta potential further changed to +23 and +20 mV, respectively (Figure 3c). These changes in the FTIR spectra and the shifts in zeta potential values provide strong evidence for the successful functionalization of the UCNPs with Van and Poly. With the antibiotics conjugated to the UCNPs, the next step was to explore how these nanoparticles interact with AuNPs to achieve the desired “turn-off” effect.

### Ratiometric Emission Quenching

By combining the functionalized UCNPs with AuNPs, we explored the luminescence quenching of the former by the surface plasmon resonance of the latter, establishing a baseline “off” state for the sensor. The quenching of UCNP emissions by AuNPs can occur via two primary mechanisms: luminescence resonance energy transfer (LRET) or the inner filter effect (IFE), both of which require spectral overlap between the UCNP emission and AuNP absorption. LRET is a distance-dependent nonradiative energy transfer process that typically results in a reduction of the donor’s (UCNP) emission lifetime, as energy is transferred to the AuNPs. In contrast, IFE arises from reabsorption or scattering of emitted photons by the AuNPs and is not dependent on nanoscale proximity. Importantly, IFE does not affect the excited-state lifetime of the donor, as it is an optical filtering phenomenon rather than a physical energy transfer.^22–24^

The AuNPs were produced using the seed-mediated method, resulting in nanoparticles with a size distribution centered at around 50 nm, as confirmed by scanning electron microscopy (SEM) analysis (Figure 4a,b). The histogram in Figure 4c represents the particle size distribution, showing a relatively narrow normal distribution. Figure 4d,e shows the normalized absorbance spectrum of the synthesized AuNPs alongside the normalized emission spectra of Er-UCNP@PEG_4_-Van and Tm-UCNP@PEG_4_-Poly, respectively. A clear spectral overlap is observed between the AuNPs’ surface plasmon resonance band (centered at 520 nm) and the UCNPs’ emission in the green (Er^3+^) and blue (Tm^3+^) regions. In contrast, the red emissions exhibit minimal overlap with AuNP absorption, suggesting a selective quenching effect. This differential overlap underpins the ratiometric sensing strategy, where quenching is more pronounced in the green and blue bands and less so in the red bands, enabling G/R and B/R ratio-based detection.

**Figure 4:**
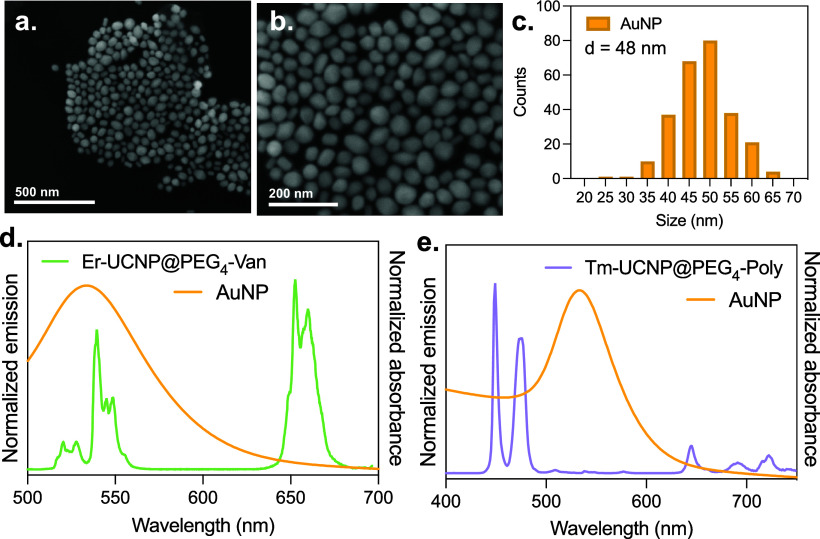
AuNP characterization. (a, b) SEM images of the synthesized AuNPs. (c) Histogram of the particle size distribution derived from the SEM images. The normalized absorbance spectrum of AuNPs and the normalized emission spectra of (d) Er-UCNP@PEG_4_-Van and (e) Tm-UCNP@PEG_4_-Poly.

#### Er-UCNP@PEG_4_-Van Emission Quenching

To make the UCNPs “turned-off”, aqueous dispersions of Er-UCNP@PEG_4_-Van were combined with increasing concentrations of AuNPs. The collected emission spectra and calculated quenching percentages are presented in Figure 5a,b, respectively. The quenching was quantified by integrating the areas under the green (500–550 nm), red (550–700 nm), and total (500–700 nm) emissions, dividing these values by the emissions of Er-UCNP@PEG_4_-Van in the absence of AuNPs, and converting these ratios to a quenching percentage.

**Figure 5:**
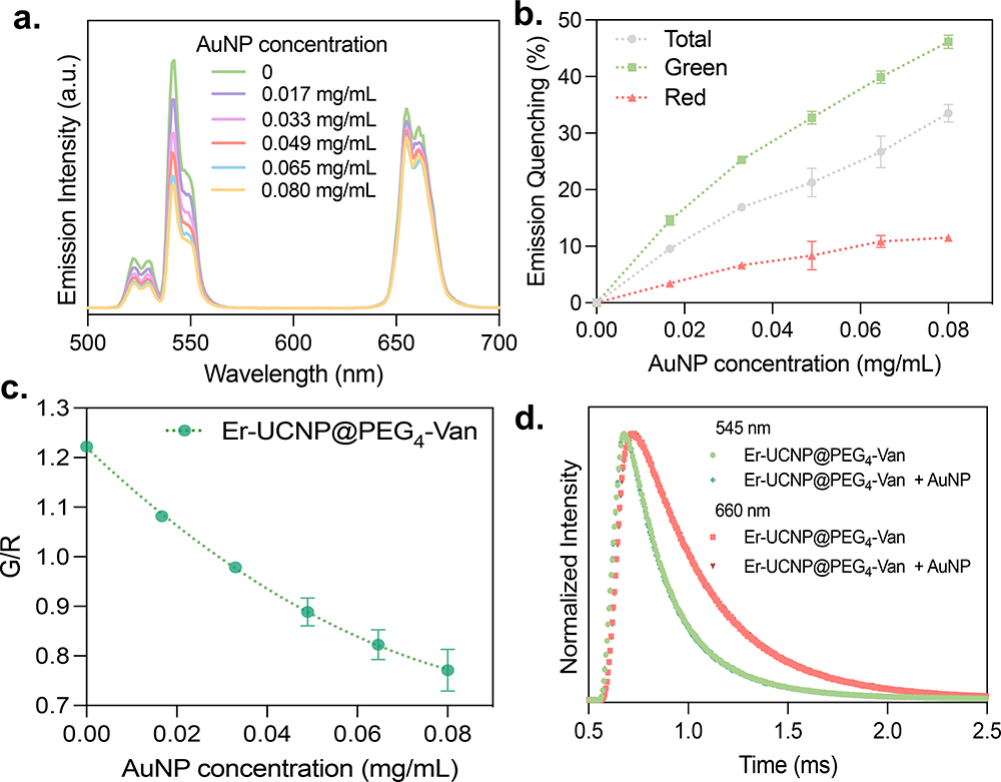
Quenching effect of AuNPs on Er-UCNPs and ratiometric analysis. (a) Emission spectra of Er-UCNP@PEG_4_-Van (0.5 mg/mL) with varying concentrations of AuNPs. (b) Quenching (%) of green, red, and total Er-UCNP@PEG_4_-Van emissions with increasing concentrations of AuNPs. (c) Plot of the green/red (G/R) emission ratio versus AuNP concentration, illustrating the quenching effect. (d) Emission lifetime at 545 and 660 nm of Er-UCNP@PEG_4_-Van (0.5 mg/mL) with and without AuNPs (0.08 mg/mL).

The results demonstrate that as the concentration of AuNPs increases, there is a marked decrease in the Er^3+^ green emission, while the red emission show a much lower degree of quenching. Specifically, at a AuNP concentration of 0.08 mg/mL, the green emission quenching reaches 46%, whereas the red and total quenching are around 11% and 33%, respectively. This high level of quenching in the green region can be attributed to the significant overlap between the UCNP emission and the AuNP absorbance. In contrast, the lower quenching in the red region is likely due to a reduced spectral overlap and possible scattering or dilution effects. Using the emission areas, the G/R ratio was calculated for each AuNP concentration, and it varied linearly from 1.22 to 0.75, as shown in Figure 5c.

By comparing the emission lifetimes before and after the introduction of AuNPs, we could discern whether LRET or IFE was the dominant process. The Er-UCNP@PEG_4_-Van 545 and 660 nm emission lifetimes were measured as 236 and 403 μs, respectively. Interestingly, after the addition of AuNPs, the values remained almost unchanged, at 241 and 407 μs for the detection of green and red emissions, respectively (Figure 5d). These results strongly suggest that the quenching is mainly due to IFE, where the luminescence of the UCNPs is reabsorbed by the AuNPs.

#### Tm-UCNP@PEG_4_-Poly Emission Quenching

The next step involved evaluating the response of Tm-UCNP@PEG_4_-Poly to the presence of AuNPs. Upon combining the nanoparticles, a higher quenching effect was observed in the blue emission of the Tm-UCNPs compared to that in the red emission, as shown in Figure 6a. The calculated quenching percentages for the blue (400–550 nm), red (550–750 nm), and total (400–750 nm) emissions were plotted against the concentration of gold nanoparticles (Figure 6b). The quenching values obtained at a AuNP concentration of 0.08 mg/mL were 37% for the blue emission, 21% for the red emission, and 31% for the total emission. The observed quenching trends can be attributed to the spectral overlap between the Tm-UCNP emissions and the AuNP absorbance band. Although the overlap is not as extensive as that observed for Er-UCNPs, the partial overlap in the blue region still results in significant quenching. The B/R exhibited a linear decrease from 2.11 to 1.70 with increasing concentrations of AuNPs, as shown in Figure 6c.

**Figure 6:**
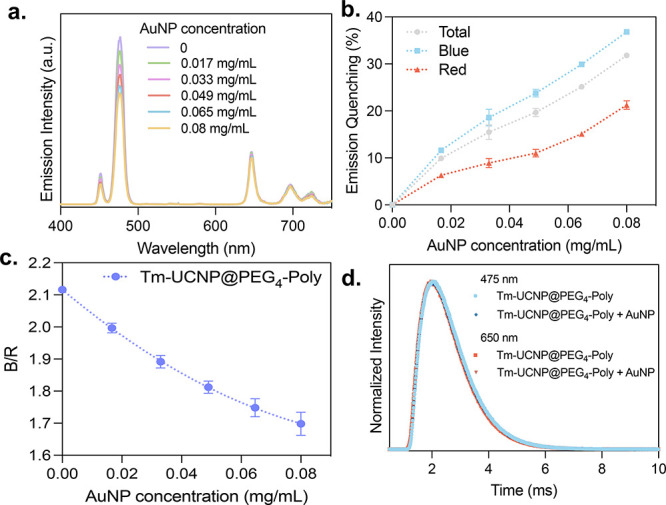
Quenching effect of AuNPs on Tm-UCNPs and ratiometric analysis. (a) Emission spectra of Tm-UCNP@PEG_4_-Poly (0.5 mg/mL) with varying concentrations of AuNPs. (b) Quenching (%) of blue, red, and total Tm-UCNP@PEG_4_-Poly emissions with increasing concentrations of AuNPs. (c) Plot of the blue/red (B/R) emission ratio versus AuNP concentration, illustrating the quenching effect. (d) Emission lifetime at 475 and 650 nm of Tm-UCNP@PEG_4_-Poly (0.5 mg/mL) with and without AuNPs (0.08 mg/mL).

No significant change in the lifetimes was detected, monitoring both blue and red emissions, with the 650 nm lifetime varying from 1.05 to 1.1 ms upon the presence of AuNPs and the 475 nm lifetime remaining at 1.1 ms before and after the addition of the metallic nanoparticles, suggesting the occurrence of IFE.

With the optimized nanoparticle systems in the “off” state, we proceeded to the bacteria detection.

### Ratiometric Bacteria Sensing

#### Gram-Positive Bacteria Sensing

The Er-UCNP@PEG_4_-Van and AuNPs (0.5 and 0.08 mg/mL, respectively) “turned-off” system was applied in the detection of *Staphylococcus aureus* (*S. aureus*), which was used as a model Gram-positive strain. The nanoparticles were combined with increasing concentrations of the bacteria in phosphate-buffered saline (PBS) at pH ∼ 7.4 and ambient temperature (∼25 °C) and incubated for 10 min to allow for binding interactions. The emission spectra were collected in triplicate for each bacterial concentration, and the integrated green and red emission areas were used to calculate the G/R ratio for each point (Figure 7a). To account for variations and provide a clearer comparison, these G/R ratios were normalized against the G/R ratio of the initial reference sample (Er-UCNP@PEG_4_-Van + AuNPs). The normalized G/R values, denoted as G/R_n_, were plotted against the bacteria concentration, as shown in Figure 7b. The G/R_n_ increases upon bacteria addition due to the partial recovery of UCNP green emission, while the red emission remains unchanged. The G/R_n_ values varied from 1.03 for 0.05 CFU/mL to 1.25 for 5 × 10^5^ CFU/mL, following a pattern that could be accurately fitted with a polynomial equation, achieving an *R* ^2^ value of 0.9885.

**Figure 7:**
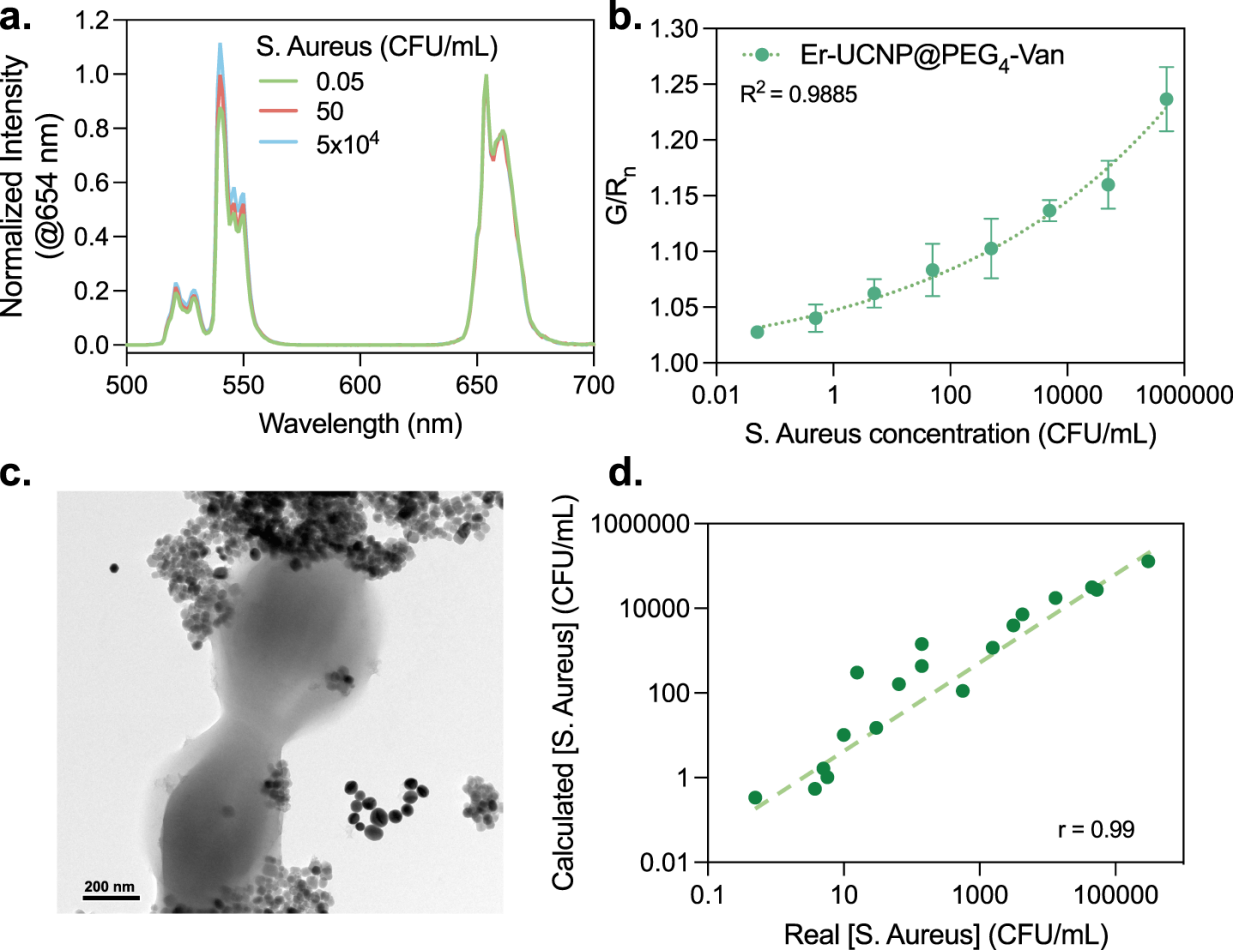
*S. aureus* detection using Er-UCNP@PEG_4_-Van and AuNPs. (a) Emission spectra of the Er-UCNP@PEG_4_-Van (0.5 mg/mL) and AuNPs (0.08 mg/mL) in the presence of increasing concentrations of *S. aureus*. (b) G/R_n_ emission ratio versus *S. aureus* concentration calibration curve (*R* ^2^ = 0.99). (c) TEM image of *Staphylococcus aureus* mixed with Er-UCNP@PEG_4_-Van and AuNPs. (d) Correlation between the calculated concentration of *S. aureus* in spiked samples versus the real concentration (Pearson *r* = 0.99).

It is important to note that the reported lowest concentration of 0.05 CFU/mL corresponds to the nominal value based on serial dilution of a standardized inoculum and may not reflect the exact number of viable bacteria present in the sample. At such low concentrations, stochastic variation in the bacterial presence and measurement noise may contribute to signal fluctuations, which could result in false positives or overestimated sensor sensitivity. Although the system demonstrated a consistent luminescent response at this concentration, we acknowledge the need for further testing with more replicates and alternative quantification methods to validate the sensor’s robustness and reliability near the detection limit. This limitation is especially relevant for real-sample applications, where sample complexity and matrix effects may further influence the sensitivity. Moreover, it is important to note that the current study was conducted under controlled buffer conditions, and future investigations should assess the influence of varying pH and ionic strength on sensor stability and performance to ensure applicability across different biological and environmental settings.

In the TEM images of the *S. aureus* strains combined with the Er-UCNP@PEG_4_-Van + AuNPs mixture (Figure 7c), it is possible to observe the Er-UCNP@PEG_4_-Van bound and agglomerated around the Gram-positive microorganisms, while the AuNPs were observed to be more isolated and detached from the bacterial cell wall. Since IFE was suggested as the quenching mechanism taking place between the UCNPs and AuNPs, we hypothesize that the partial recovery in UCNP emission upon bacterial binding results from the physical separation of UCNPs and AuNPs driven by the formation of a bacteria–nanoprobe complex. This separation reduces the optical density in the immediate environment of the UCNPs, thereby weakening the inner filter effect and allowing more of the UCNP emission to be detected. Additionally, steric hindrance and surface crowding from bacterial attachment may limit further AuNP proximity to the UCNPs, reinforcing the reduced quenching. Although the signal modulation is modest, the use of a ratiometric approach provides enhanced reliability by internally normalizing emission fluctuations, allowing for consistent and reproducible sensing performance under controlled conditions.

We used the developed sensor to quantify *S. aureus* in 20 spiked samples, and the concentrations calculated using the G/R_n_ presented a good correlation with the real bacteria concentrations (with the Pearson correlation coefficient (*r*) = 0.99), as shown in Figure 7d. The obtained results demonstrate the ability of Er-UCNP@PEG_4_-Van to target Gram-positive bacteria and the clear relationship between the *S. aureus* concentration and the G/R_n_ values, indicating the sensor’s ability to detect and quantify its presence through the ratiometric change in UCNP emission.

#### Gram-Negative Bacteria Sensing

Following the successful detection of *S. aureus* using the Er-UCNP@PEG_4_-Van system, the Tm-UCNP@PEG_4_-Poly + AuNPs (0.5 and 0.08 mg/mL, respectively) system was then tested to detect *Escherichia coli* (*E. coli*), a model Gram-negative bacterium. The nanoparticles were mixed with varying concentrations of *E. coli* in PBS at pH ∼ 7.4 and ambient temperature (∼25 °C) and incubated for 10 min to allow for binding interactions. The emission spectra were recorded for each bacterial concentration (Figure 8a), focusing on the integrated blue and red emission areas to calculate the B/R ratio. To ensure consistency and an accurate comparison, the B/R ratios were normalized relative to the initial reference sample (Tm-UCNP@PEG_4_-Poly + AuNPs).

**Figure 8:**
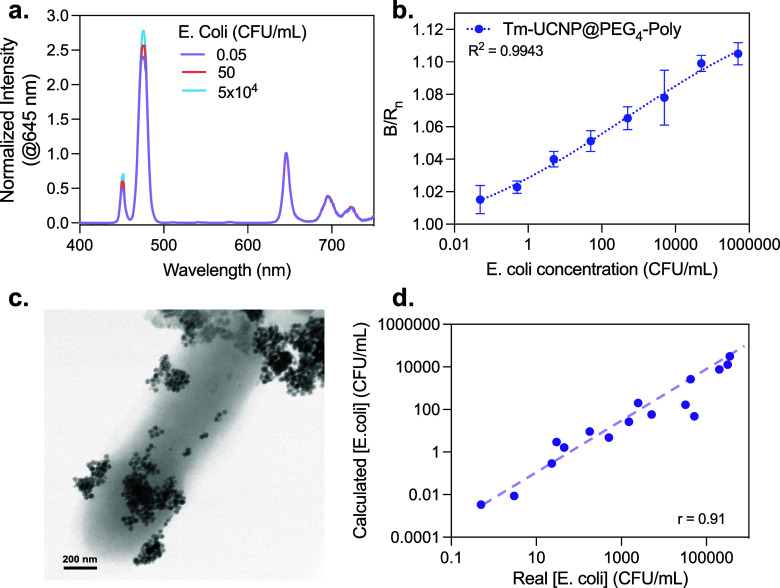
*E. coli* detection using antibiotic-functionalized UCNPs and AuNPs. (a) Emission spectra of the Tm-UCNP@PEG_4_-Poly (0.5 mg/mL) and AuNPs (0.08 mg/mL) in the presence of increasing concentrations of *E. coli*. (b) B/R_n_ emission ratio versus *E. coli* concentration calibration curve (*R* ^2^ = 0.99). (c) TEM image of *Escherichia coli* mixed with Tm-UCNP@PEG_4_-Poly and AuNPs. (d) Correlation between the calculated concentration of *E. coli* in spiked samples versus the real concentration (Pearson *r* = 0.91).

The normalized B/R values, denoted as B/R_n_, were plotted against the bacteria concentration (Figure 8b). The introduction of bacteria increased the B/R_n_, attributed to the partial recovery of the UCNP blue emission, while the red emission remained relatively stable. The B/R_n_ values spanned from 1.02 at 0.05 CFU/mL to 1.11 at 5 × 10^5^ CFU/mL, and the B/R_n_ versus *E. coli* concentration pattern was modeled with a polynomial equation, yielding an *R* ^2^ value of 0.9943.

In the TEM images of the *E. coli* combined with the Tm-UCNP@PEG_4_-Poly + AuNPs mixture (Figure 8c), Tm-UCNP@PEG_4_-Poly was visibly bound and clustered around the Gram-negative bacteria, while the AuNPs appeared more dispersed and not attached to the bacterial cell wall.

Using the developed sensor, we quantified *E. coli* in 20 spiked samples, and the concentrations calculated using the B/R_n_ showed a strong correlation with the actual bacterial concentrations (*r* = 0.91, Figure 8d). These results demonstrate the effectiveness of Tm-UCNP@PEG_4_-Poly in targeting Gram-negative bacteria and confirm the clear relationship between the *E. coli* concentration and B/R_n_ values.

#### Gram-Positive and Gram-Negative Bacteria Sensing

We also evaluated the dual-mode detection capability of our sensor by testing a mixture of Er-UCNP@PEG_4_-Van and Tm-UCNP@PEG_4_-Poly to detect, differentiate, and quantify the bacteria. Specifically, we mixed Er-UCNP@PEG_4_-Van (0.15 mg/mL) and Tm-UCNP@PEG_4_-Poly (0.35 mg/mL) with AuNPs (0.08 mg/mL) to have the initial “turned-off” system.

Upon the presence of increasing concentrations of *S. aureus* (Figure 9a,b), there is a clear increase in the G/R_n_ ratio, indicating that the green emission from the Er-UCNPs partially recovers as the bacteria bind to the UCNPs, suggesting effective targeting and detection of Gram-positive bacteria by the Er-UCNP@PEG_4_-Van system. Conversely, the B/R_n_ ratio remains almost unchanged, showing that the Tm-UCNP@PEG_4_-Poly emissions are less affected by the presence of *S. aureus*.

**Figure 9:**
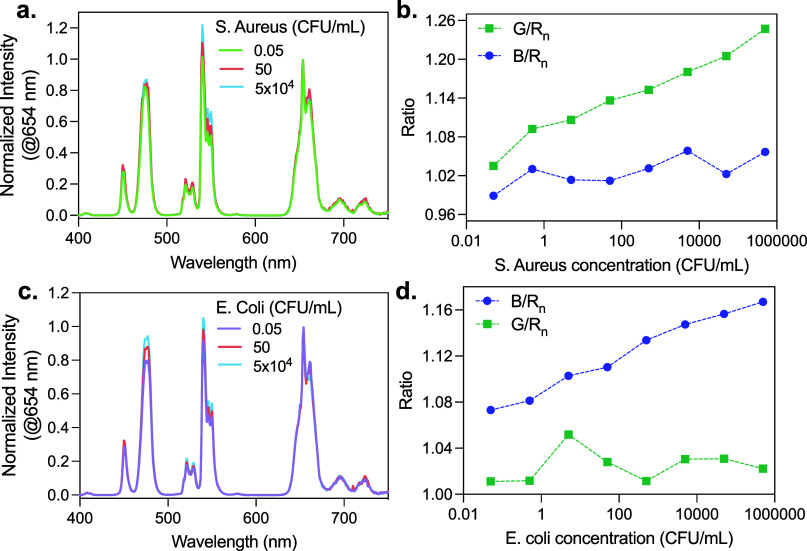
Detection of *Staphylococcus aureus* and *Escherichia coli* using a mixture of Er-UCNP@PEG_4_-Van, Tm-UCNP@PEG_4_-Poly, and AuNPs. (a) Emission spectra of the nanoparticle mixture (Er-UCNP@PEG_4_-Van, 0.15 mg/mL; Tm-UCNP@PEG_4_-Poly, 0.35 mg/mL; and AuNPs, 0.08 mg/mL) with increasing concentrations of *S. aureus.* (b) The normalized G/R_n_ and B/R_n_ ratios plotted against *S. aureus* concentration. (c) Emission spectra of the same nanoparticle mixture with increasing concentrations of *E. coli.* (d) The normalized G/R_n_ and B/R_n_ ratios plotted against *E. coli* concentration.

In contrast, when the same nanoparticle mixture is exposed to *E. coli* (Figure 9c,d), there is a significant increase in the B/R_n_ ratio, reflecting the partial recovery of the blue emission from the Tm-UCNP@PEG_4_-Poly. This indicates that the Tm-UCNP@PEG_4_-Poly system effectively targets Gram-negative bacteria. Meanwhile, the G/R_n_ ratio shows minimal change, suggesting that Er-UCNP@PEG_4_-Van is less responsive to *E. coli*.

These observations confirm that the mixture of Er-UCNP@PEG_4_-Van and Tm-UCNP@PEG_4_-Poly can successfully differentiate between Gram-positive and Gram-negative bacteria by selectively restoring emissions in response to bacterial binding. The control experiments are presented in the Supporting Information, further validating the sensor’s specificity and confirming that the observed changes in G/R_n_ and B/R_n_ are indeed due to the presence of the target bacteria and not caused by nonspecific interactions or other environmental factors. This dual-mode detection system offers a novel and reliable approach to bacterial detection, differentiation, and quantification, demonstrating its potential for practical applications.

Despite the promising results demonstrated in this study, several limitations must be acknowledged. First, the sensor was evaluated using only two representative bacterial strains, which limits conclusions regarding its broad-spectrum applicability. Second, the signal modulation relies on a relatively modest emission recovery due to the inner filter effect, which may constrain the sensitivity under certain conditions. Third, the experiments were conducted in buffered laboratory media, and the sensor’s performance in complex real-world samples remains to be determined. Lastly, while TEM images qualitatively confirmed nanoparticle–bacteria association, higher-resolution or element-specific imaging techniques would be needed to fully characterize the nanoscale interactions. Addressing these aspects in future studies will be essential for translating this proof-of-concept into practical applications.

## Conclusion

We successfully developed an innovative UCNP-based luminescent sensor capable of detecting, differentiating, and quantifying Gram-positive and Gram-negative bacteria in solution. By functionalizing Er^3+^- and Tm^3+^-doped UCNPs with vancomycin and polymyxin-B, respectively, and employing gold nanoparticles as quenching agents, we established a ratiometric “turn-on” sensing mechanism based on the selective recovery of UCNP emission upon bacterial binding. The sensor design effectively utilized variations in the G/R and B/R emission ratios to differentiate between *Staphylococcus aureus* (Gram-positive) and *Escherichia coli* (Gram-negative), respectively. It operated over a broad concentration range (0.05 to 5 × 10^5^ CFU/mL) and demonstrated strong linear correlations between luminescent signal and bacterial concentration (*r* = 0.99 for *S. aureus*, *r* = 0.91 for *E. coli*), underscoring its potential for accurate and quantitative detection. However, as a proof-of-concept, this study presents limitations. Most notably, only two representative bacterial strains were evaluated. To validate the platform’s broader applicability and specificity, future work should focus on testing a wider range of clinically and environmentally relevant Gram-positive and Gram-negative species, including drug-resistant strains. Additional efforts should include testing in complex real-world samples and employing advanced characterization techniques to better understand nanoparticle–bacteria interactions. With these limitations in mind, the promising results presented here lay the foundation for the development of practical, real-world UCNP-based biosensors for bacterial detection and differentiation.

## Materials and Methods

### Synthesis of β-UCNPs (NaYF_4_:18%Yb^3+^,2%Er^3+^/25%Yb^3+^,0.3%Tm^3+^)

UCNPs with a hexagonal structure were synthesized following a high-temperature coprecipitation method as previously reported.^25^ LnCl_3_ aqueous solutions [0.78 mL of YCl_3_ (1 M), 0.20 mL of YbCl_3_ (1 M), and 0.20 mL of ErCl_3_ (0.1 M)] were transferred to a 100 mL three-necked round-bottom flask and heated to evaporate water. The resulting powder was mixed with 6 mL of oleic acid (OA) and 15 mL of octadecene (ODE), heated to 150 °C for 30 min under a nitrogen or argon atmosphere to form a homogeneous solution, and then cooled to room temperature. Next, 5 mL of a methanol solution containing NaOH (0.1 g) and NH_4_F (0.148 g) was slowly added into the flask, forming solid-state precipitates in the solution. Subsequently, the solution was slowly heated to 110 °C to evaporate methanol, degassed for 10 min, and then heated to 300 °C and maintained for 1 h under an inert atmosphere. After the solution was naturally cooled down, nanocrystals were precipitated with acetone, isolated via centrifugation (6000 rpm, 10 min), and washed once with acetone and twice with ethanol. NaYF_4_:25%Yb,0.3%Tm nanocrystals were also synthesized by changing the molar ratio of the reagents (YCl_3_:YbCl_3_:TmCl_3_) to 74.7:25:0.3.

### Coating UCNPs with a Shell of the Undoped Matrix (NaYF_4_:Yb,Er/Tm@NaYF_4_ = Er/Tm-UCNPs)

The procedure to coat the nanoparticles with an inert matrix shell was similar to that used for the synthesis of core UCNPs. First, 1 mL of YCl_3_ aqueous solution (1 M) was transferred to a 100 mL three-necked round-bottom flask and heated until dried. The resulting powder was mixed with 6 mL of OA and 15 mL of ODE and heated to 150 °C for 30 min to form a yellow homogeneous and clear solution. After cooling to room temperature, the as-prepared UCNPs (redispersed in 15 mL of cyclohexane) were added to the above solution, and the mixture was heated to 100 °C. After cyclohexane was removed, the synthesis proceeded following the same steps as those of NaYF_4_:Yb,Er/Tm nanoparticles. The final core–shell nanocrystals were washed with acetone one time and with ethanol two times and dried at room temperature.

### Ligand Exchange of Oleic Acid for Poly­(ethylene glycol) Diacid (UCNP@PEG_4_-COOH)

For the ligand exchange reaction, 25 mg of UCNP@OA was dispersed in 12 mL of distilled water containing 35 mg of COOH-PEG_4_-COOH, and the reaction was kept under sonication for 3 h. Subsequently, the dispersion was extracted twice with diethyl ether to remove the oleic acid, and the nanoparticles were finally isolated by centrifugation (6000 rpm, 40 min), washed 2 times with ethanol, and dried at room temperature. The same reaction was realized for Er- and Tm-doped UCNPs.

### Antibiotic Addition to UCNP@PEG_4_-COOH (Er-UCNP@PEG_4_-Van/Tm- UCNP@PEG_4_-Poly)

#### UCNP Activation

5 mg of UCNP@PEG_4_-COOH was dispersed in 5 mL of 30 mM MES buffer (pH = 5.5) and mixed with 4 mg of EDC and 3.6 mg of NHS. The reaction was kept under stirring at room temperature for at least 2 h before further use.

#### UCNP Functionalization

15 mg of antibiotic (Van for Er-UCNPs or Poly for Tm-UCNPs) was dispersed in 5 mL of 30 mM MES buffer (pH = 5.5) and then transferred to the flask containing the activated nanoparticles. The reaction was allowed to proceed at room temperature overnight, and then the nanoparticles were collected by centrifugation (6000 rpm, 40 min), washed 2 times with MES buffer, and dried.

### Gold Nanoparticle (AuNP) Synthesis

The gold nanoparticles (AuNPs) were synthesized by using a citrate reduction method. First, a 10 mL solution of 1 mM HAuCl_4_ was prepared and heated to boiling under constant stirring. Once the solution reached its boiling point, 1 mL of 0.06 M sodium citrate (Na_3_Cit) solution (15.5 mg/mL) was rapidly added to the boiling solution. The mixture was then allowed to reflux for 15 min, during which time the color of the solution changed, indicating the formation of AuNPs. After the reflux period, the solution was removed from heat and allowed to cool naturally to room temperature. The resulting AuNPs were then stored at room temperature for future use.^26^

### Quenching Experiments: Combining UCNPs and AuNPs

For the UC emission quenching experiments, the UCNPs were combined with different concentrations of AuNPs (0.008–0.08 mg/mL). After each AuNP addition, the emission spectra were collected in triplicate. The mean values between the three spectra were calculated, and the obtained spectra were integrated. For Er- and Tm-UCNPs, the spectra were collected between 500 and 700 nm and between 400 and 750 nm, respectively, with 980 nm excitation. The results were analyzed in terms of whole (all), green (500–550 nm for Er-UCNPs), blue (400–550 nm for Tm-UCNPs), and red (550–750/750 nm) emissions. The integrated areas were normalized by the emission of the UCNPs in the absence of gold nanostructures, and the quenching percentage was calculated as (1 – NA) × 100, where NA = normalized area. The integrated areas were also used to calculate the green/red (G/R) and blue/red (B/R) ratios for each bacterial concentration.

The G/R ratio was calculated using the integrated emission areas according to the equation G/R = *A*_green_/*A*_red_, where *A*_green_ is the integrated intensity from 500–550 nm and *A*_red_ is from 550–700 nm. Similarly, for Tm-UCNPs, the B/R ratio was calculated as B/R = *A*_blue_/*A*_red_, where *A*_blue_ corresponds to 400–550 nm and *A*_red_ to 550–750 nm.

### Bacterial Culture and Inoculum Preparation

The bacterial strains used in this study were *Escherichia coli* (ATCC 25922) and *Staphylococcus aureus* (ATCC 25923). For cultivation, 1 mL of frozen bacterial stock (stored in Brain Heart Infusion [BHI] medium with 20% glycerol) was transferred to 9 mL of fresh BHI medium. The cultures were incubated overnight at 37 °C with shaking at 150 rpm in a shaker incubator. The bacterial inocula were standardized to 10^8^ colony-forming units per milliliter (CFU/mL) based on optical density measurements at 600 nm (OD_600_). Following standardization, the bacterial suspensions were centrifuged twice at 4000 rpm for 10 min each. After each centrifugation, the supernatant was discarded.

### Bacteria Sensing Assays

After the ideal concentrations of UCNPs and AuNPs for the sensor were determined, increasing concentrations of bacteria (5 to 5 × 10^5^ CFU/mL) were added to the mixtures to analyze their effect on the UCNPs’ emission. For Er-UCNP@PEG_4_-Van, *Staphylococcus aureus* (*S. aureus*) was used as a Gram-positive model, and *Escherichia coli* (*E. coli*) was used as a Gram-negative model combined with Tm-UCNPs@PEG_4_-Poly. The bacteria were mixed with the UCNPs–AuNPs pairs in aqueous media, and the system was incubated for 10 min. As in the quenching experiments, the emission spectra were collected in triplicate for each bacterial concentration, and the mean values were used in the next steps. For Er- and Tm-UCNPs, the spectra were collected between 500 and 700 nm and between 400 and 750 nm, respectively, with 980 nm excitation. The results were analyzed in terms of whole (total), green (500–550 nm, for Er-UCNPs), blue (400–550 nm, for Tm-UCNPs), and red (550–700/750 nm) emissions. The ratios between the integrated areas were divided by the emission ratios of the initial system (UCNPs–AuNPs in the absence of bacteria), and the normalized green/red (G/R_n_) and blue/red (B/R_n_) ratios were plotted against bacteria concentration.

## Supplementary Material



## Notes

Control experiments for assessing the dilution effect, the antibiotic specificity for target bacteria, the role of AuNPs as quenchers, and the nanoparticle specificity to target bacteria (PDF)
