# Supplementary material for: A Dual-Mode “Turn-On” Ratiometric Luminescent Sensor Based on Upconverting Nanoparticles for Detection and Differentiation of Gram-Positive and Gram-Negative Bacteria
Source: ACS Omega. 2025 Sep 23;10(39):46040–50. doi: 10.1021/acsomega.5c07006 (PMC12509115; doi:10.1021/acsomega.5c07006)
Supplement: Supplementary file 1 [file ao5c07006_si_001.pdf]

SUPPORTING INFORMATION

A Dual-Mode “Turn-on” Ratiometric Luminescent Sensor Based on Upconverting Nanoparticles for Detection and Differentiation of Gram-Positive and Gram-Negative Bacteria

Marylyn S. Arai[1], Gabriel V. Brambilla[1], Bruna Carolina Corrêa[1], Leonnam Merízio[1], Natália M. Inada[1], Andrea S. S. de Camargo[2,3]

[1] São Carlos Institute of Physics, University of São Paulo,13566-590, São Carlos, Brazil
[2] Federal Institute for Materials Research and Testing (BAM), 12489 Berlin, Germany
[3] Friedrich-Schiller University (FSU), 07743, Jena, Germany

[*] Corresponding authors
Andrea S. S. de Camargo
andrea.camargo@bam.de

Marylyn S. Arai
marylyn.setsuko@gmail.com

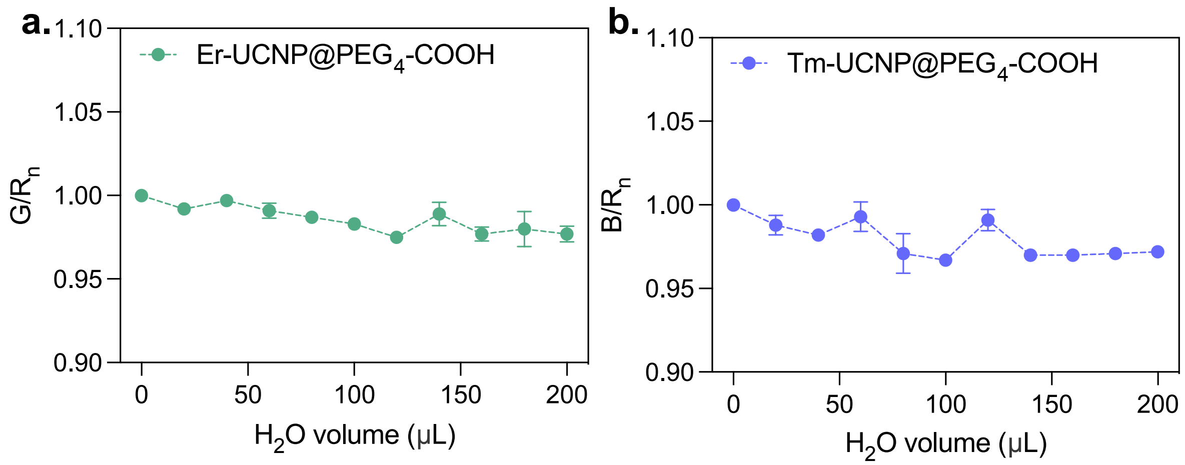

Figure S1. Control Experiment 1 – Assessing the Dilution Effect. a. G/R_n ratio vs. added H_2O volume for Er-UCNP@PEG$_4$-COOH**. b.** B/R_n ratio vs. added H_2O volume for Tm-UCNP@PEG$_4$-COOH. Increasing volumes of H_2O were added to a 2 mL dispersion of 0.5 mg/mL UCNPs. The results indicate that dilution does not cause significant changes in the emission ratios, confirming that the observed changes in G/R_n and B/R_n are due to the presence of AuNPs.

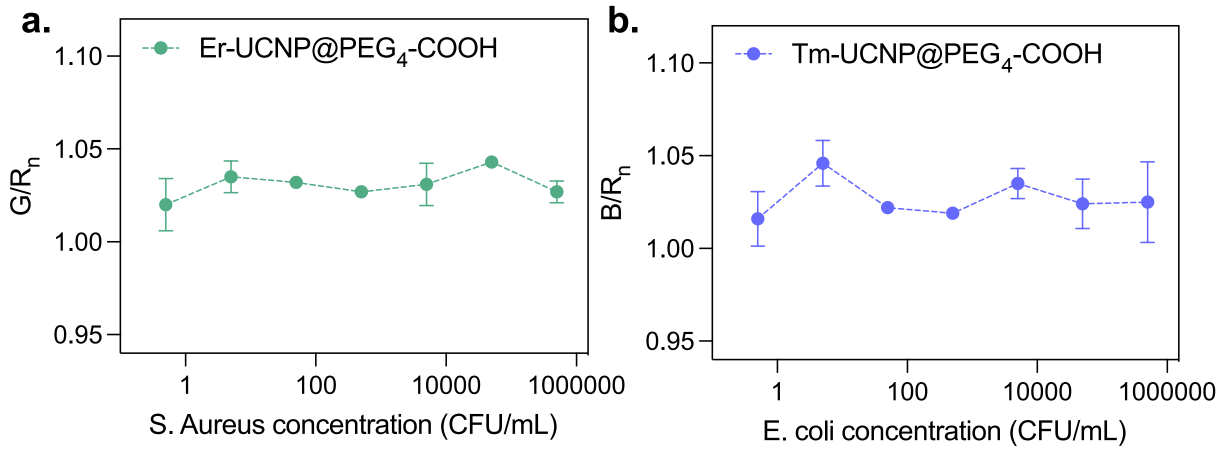

Figure S2. Control Experiment 2 – Antibiotic Specificity for Target Bacteria. a. G/R_n ratio vs. S. aureus concentration for Er-UCNP@PEG$_4$-COOH. **b.** B/R_n ratio vs. E. coli concentration for Tm-UCNP@PEG$_4$-COOH. UCNP@PEG$_4$-COOH (0.5 mg/mL) without antibiotics was combined with AuNPs (0.08 mg/mL) and incubated with increasing bacterial concentrations. The results show that G/R_n and B/R_n do not change significantly upon microorganism addition, indicating that the antibiotics are responsible for specific targeting and the observed changes in emission ratios.

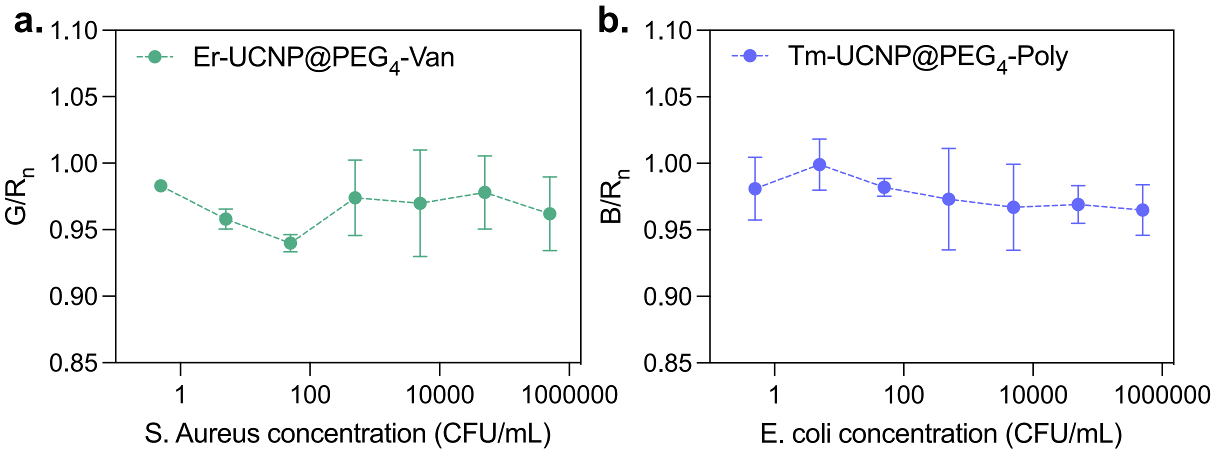

Figure S3. Control Experiment 3 – Role of AuNPs as Quenchers. a. G/R_n ratio vs. S. aureus concentration for Er-UCNP@PEG_4-Van. **b.** B/R_n ratio vs. E. coli concentration for Tm-UCNP@PEG_4-Poly. Antibiotic-functionalized UCNPs (0.5 mg/mL) were incubated with increasing concentrations of bacteria in the absence of AuNPs. The presence of bacteria alone does not cause significant changes in G/R_n and B/R_n, indicating that the observed emission changes in the sensor require AuNPs and are due to the diminishing inner filter effect.

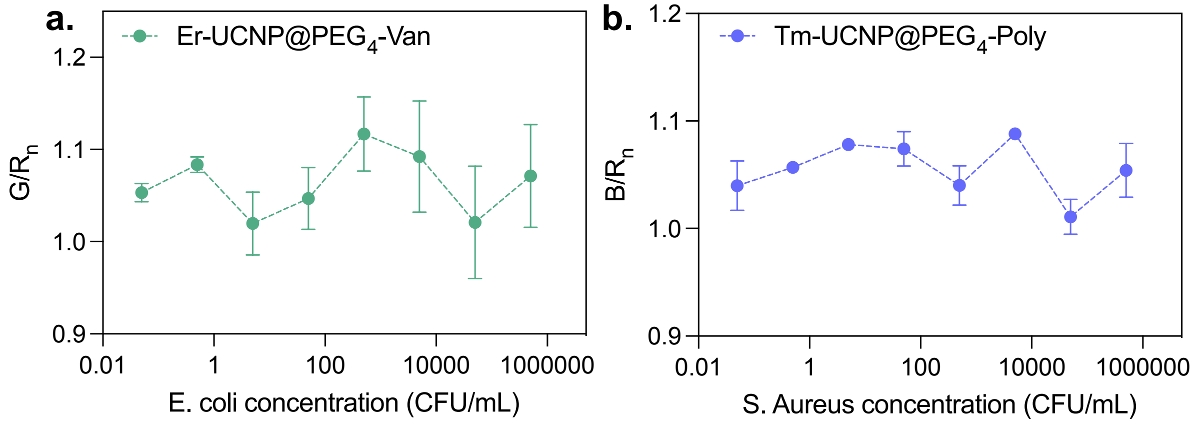

Figure S4. Control Experiment 4 – Nanoparticle Specificity to Target Bacteria. a. G/R_n ratio vs. E. coli concentration for Er-UCNP@PEG_4-Van. **b.** B/R_n ratio vs. S. aureus concentration for Tm-UCNP@PEG_4-Poly. Antibiotic-functionalized UCNPs (0.5 mg/mL) were combined with AuNPs (0.08 mg/mL) and incubated with increasing bacterial concentrations. Er-UCNP@PEG_4-Van was tested with E. coli and Tm-UCNP@PEG_4-Poly with S. aureus. No trend in emission ratios was detected with increasing microorganism concentration, indicating that Er-UCNP@PEG_4-Van and Tm-UCNP@PEG_4-Poly selectively target Gram-positive and Gram-negative bacteria, respectively.
